# Changes of protein levels in human urine reflect the dysregulation of signaling pathways of chronic kidney disease and its complications

**DOI:** 10.1038/s41598-020-77916-z

**Published:** 2020-11-27

**Authors:** Yiming Hao, Luis Tanon Reyes, Robert Morris, Yifeng Xu, Yiqin Wang, Feng Cheng

**Affiliations:** 1 Shanghai Key Laboratory of Health Identification and Assessment/Laboratory of TCM Four Diagnostic Information, Shanghai University of Traditional Chinese Medicine, Shanghai, 201203 China; 2 Department of Pharmaceutical Sciences, College of Pharmacy, University of South Florida, Tampa, FL 33612 USA

**Keywords:** Systems biology, Nephrology

## Abstract

The increasing prevalence of chronic kidney disease (CKD) seriously is threatening human health and overall quality of life. The discovery of biomarkers of pathogenesis of CKD and the associated complications are very important for CDK diagnosis and treatment. In this paper, urine protein biomarkers were investigated because urine sample collection is convenient and non-invasive. We analyzed the protein concentrations in the urine of CKD patients and extracted abnormal protein signals comparing with the healthy control groups. The enriched signaling pathways that may characterize CKD pathology were identified from these proteins. We applied surface-enhanced laser desorption and ionization time of flight mass spectrometry technology to detect different protein peaks in urine samples from patients with CKD and healthy controls. We searched the proteins corresponding to protein peaks through the UniProt database and identified the signaling pathways of CKD and its complications by using the NIH DAVID database. 42 low abundance proteins and 46 high abundance proteins in the urine samples from CKD patients were found by comparing with healthy controls. Seven KEGG pathways related to CKD and its complications were identified from the regulated proteins. These pathways included chemokine signaling pathway, cytokine–cytokine receptor interaction, oxidative phosphorylation, cardiac muscle contraction, Alzheimer’s disease, Parkinson's disease, and salivary secretion. In CKD stages 2, 3, 4, and 5, five proteins showed significantly differential abundances. The differential protein signals and regulated signaling pathways will provide new insight for the pathogenesis of CKD and its complications. These altered proteins may also be used as novel biomarkers for the noninvasive and convenient diagnosis methods of CKD and its complications through urine testing in the future.

## Introduction

Chronic kidney disease (CKD) is a disease characterized by progressive loss of renal function over extended periods of time, of which spans from months to years. Its prevalence is increasing dramatically, and it threatens human health and quality of life. Currently, about 8–10% of the world's population has a certain degree of CKD and is the 16th most frequent cause of mortality each year^1,2^. Unfortunately, the increasing cost of treating CKD and its complications has placed a considerable economic burden on health care systems worldwide^3^. However, in the early and middle stages of CKD, patients generally do not exhibit any noticeable signs or symptoms^1^. The current diagnostic biomarkers (serum creatinine and urine albumin) are not enough to predict the development of CKD. Therefore, timely monitoring and diagnosis of CKD is important so that patients will fare better with treatment and better outcomes will be achieved. In recent years, using human urine samples to look for biomarkers have attracted more and more attention because of its convenient, non-invasive collection, and stable composition^4^. For example, some researchers indicated that some proteins in human urine may help to predict early-stage CKD patients^5^. However, with the progression of CKD, some complications may appear gradually. Because of the complicated interplay between CKD and other comorbid conditions^1^, it is difficult to monitor the evolution of CKD by using protein concentration in human urine and thus, clinical timing of CDK diagnosis is crucial in reducing the risk of deterioration of the patient's condition. Therefore, we hope to initially understand which proteins in the urine of CKD patients may have abnormal change as well as what kind of adverse events may result from these fluctuations.

In this paper, we detected the different protein peaks existing in the samples of patients with CKD compared with healthy controls using surface enhanced laser desorption and ionization time of flight mass spectrometry (SELDI-TOF-MS) technology. Afterwards, we used proteomics databases to scan the proteins corresponding to the protein peaks observed. Finally, we explored the abnormal signaling pathways that may occur in CKD patients, as well as the related proteins and their functions that express changes in these pathways.

## Materials and methods

### Samples

CKD patients were selected from Longhua Hospital affiliated with Shanghai University of Traditional Chinese Medicine from 2008 to 2010. The cause of CKD of patients was primary chronic glomerulonephritis. One hundred and forty-seven urine samples were collected from these patients, of whom are receiving routine treatment of western medicine and traditional Chinese medicine.

The control group was selected from the healthy staff of Shanghai University of Traditional Chinese Medicine who had normal indicators of kidney function (including eGFR and SCr) and no other organic diseases. Twenty-four urine samples were collected from them.

From Table 1, there was no significant difference (*P* > 0.05) between the CKD group and healthy control group with respect to gender and age, so the data from these groups were comparable. The eGFR value of the CKD group was significantly lower than that of the healthy control group (*P* < 0.05) while SCr levels of the CKD patients were significantly higher than that of the healthy person (*P* < 0.05). In addition, among these CKD patients, there were 45 persons with stage 3, 36 individuals that had progressed to stage 4, 35 people diagnosed at stage 5, 26 with stage 2 CKD, and 5 patients with stage 1 CKD. Some of these CKD patients also had one or more secondary complications. Among them, sixty-two persons had high blood pressure, fifteen patients had heart disease, three individuals had type 2 diabetes, and one person had Alzheimer's disease.

**Table 1:** Summary of demographics and clinical information of the participants.

Demographics and clinical information	CKD group	Healthy control group
Samples number	147	24
Samples number (percentage) of stage 1	5 (3.40%)	N/A
Samples number (percentage) of stage 2	26 (17.69%)	N/A
Samples number (percentage) of stage 3	45 (30.61%)	N/A
Samples number (percentage) of stage 4	36 (24.49%)	N/A
Samples number (percentage) of stage 5	35 (23.81%)	N/A
Ratio of male to female	1:0.71	1:0.85
Average age (years)	53.64 ± 15.91	49.08 ± 11.60
eGFR (mL/min)	37.36 ± 28.27	116.67 ± 14.15
SCr (μmol/L)	264.24 ± 216.04	63.83 ± 11.24
Number (percentage) of samples with high blood pressure	62 (42.18%)	N/A
Number (percentage) of samples with heart disease	15 (10.20%)	N/A
Number (percentage) of samples with type 2 diabetes	3 (2.04%)	N/A
Number (percentage) of samples with Alzheimer's disease	1 (0.68%)	N/A

N/A: not applicable.

### Ethics approval

The study was approved by the Ethics Committee of Shanghai University of Traditional Chinese Medicine in China in 2008 and performed in accordance with the Declaration of Helsinki. All the subjects got written informed consent.

### The criteria for inclusion, exclusion, and elimination

The inclusion criteria of CKD patients include:

1. Patients accord with the diagnostic criteria of CKD which are referenced from “KDIGO 2012 clinical practice guideline for the evaluation and management of chronic kidney disease”^6^.
2. Patients’ age ranges from 20 to 85.

The exclusion criteria of CKD patients include:

1. Patients with primary heart, liver, lung, endocrine, blood, metabolism diseases, severe gastro-intestine primary diseases or mental illness.
2. Patients with CKD caused by extra-renal diseases.
3. Patients with CKD and dialysis therapy is required.
4. Patients who are pregnant or breastfeeding.
5. Patients with allergic constitution or with drug allergy.

The elimination criteria include:

Patients without complete clinical data; either due to incomplete collection or data missing.

### Experimental methods

5 mL of morning urine from each subject was collected and placed into sterilized EP tubes. The protein concentration of each urine sample was detected by ultraviolet spectrophotometry. The sample with the lowest concentration was used as the reference, and the sample with higher concentrations were diluted with stroke-physiological saline solution to reach the minimum concentration. At 4 °C, we used a centrifuge (Allegra 21R, Beckman, Inc.) to remove the impurities in the urine samples at 3000 rpm/min for 10 min, and then took the supernatant.

The unpooled urine samples were analyzed independently. They were added to an H4 protein chip (Ciphergen Biosystems, Inc.). The chip was placed in the SELDI-TOF mass spectrometer (PBSII, Ciphergen Biosystems, Inc.). The instrument was calibrated to the range of 0.1% by H4 chip with all-in-one standard protein. The parameters of mass spectrometer were as follows: laser intensity was 155, detector sensitivity was 7, the protein peaks with signal-to-noise ratio (S/N) over 5 and frequencies over 10% in all mass spectra were effective. The optimum molecular weight (Mw) ranged from 1000 to 20,000. The highest molecular weight was 40,000. Each point on the chip was collected 130 times. Data were collected with Ciphergen Protein Chip software version 3.1.1.

### Data processing, statistical analysis and substance identification

We used the total ion current to correct all the atlases to reduce the experimental errors caused by different chip and instrumental state fluctuations. The non-parametric methods including the Mann–Whitney U test (for two groups) and the Kruskal–Wallis one-way ANOVA test (for more than two groups) were used to analyze the different protein peaks. The peaks with *P* value lower than 0.05 were considered significant differences. Then we retrieved the corresponding proteins and their respective functions which were closest matched to the theoretical protein Mw value, which was according to the significantly different protein peaks, from UniProt database by using the TagIdent tool (https://web.expasy.org/tagident/). Finally, signaling pathways of CKD and its complications according to the genes corresponding to the searched proteins were analyzed by using the DAVID Bioinformatics Resources 6.8 database (https://david.ncifcrf.gov/tools.jsp). Pathways with *P* value lower than 0.05 were chosen as the possible pathways related to CKD and its complications.

## Results

**Figure 1:**
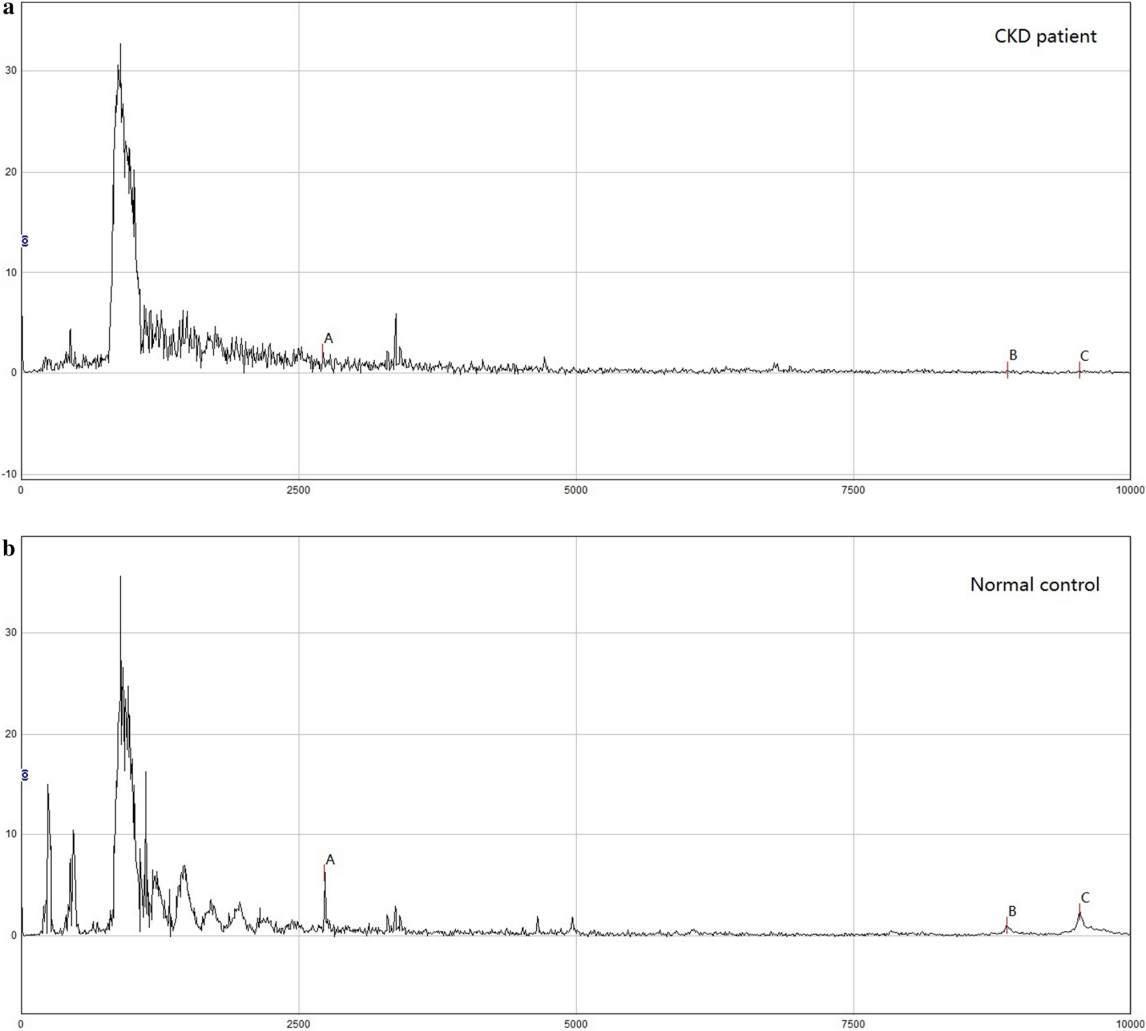
A part of protein peaks with significant differences between CKD patient and healthy control person. We compare the urine protein mass spectrum of a CKD patient with that of a healthy control person, and find three protein peaks with significant differences. All of the three peaks of CKD patients show low abundance. By retrieving the UniProt database, we suggest that peak A may be human like 11 (Mw is 2737.31 Da), peak B may be platelet basic protein (Mw is 8867.85 Da), and peak C may be high mobility group nucleus binding domain containing protein 4 (Mw is 9536.92 Da). It is suggested that the protein PPBP corresponding to platelet basic protein is involved in chemokine signaling and cytokine–cytokine receptor interaction pathway by searching the DAVID database.

We used SELDI-TOF-MS to examine urine samples from the patients with CKD and healthy control subjects. Figure 1 is a mass spectrum of a single sample comparison between two groups. As shown in Table S1, two hundred and forty-seven significantly different protein peaks were observed in the urine of patients with CKD compared with healthy controls (*P* < 0.05). In the CKD group, the abundance of one hundred and twenty-five protein peaks were significantly increased, and the abundance of one hundred and twenty-two protein peaks were significantly decreased. As listed in Table S2, eighty-eight significantly different protein peaks were found to have matched proteins in the UniProt database. Moreover, in these eighty-eight protein peaks, forty-two protein peaks that showed low abundance compared with the healthy control group, while forty-six protein peaks showed high abundance compared with the healthy controls.

Seven possible signaling pathways related to CKD and its complications were found from the identified significantly differential proteins by enrichment analysis using the NIH DAVID database. As shown in Table 2, there were three proteins included COX7B, COX6A2, and UQCRQ associated with the following pathways: oxidative phosphorylation (*P* = 0.003), cardiac muscle contraction (*P* = 0.047), Alzheimer's disease (*P* = 0.042), and Parkinson's disease (*P* = 0.028). In addition, ATP6V1G3 and COX17 were found to be involved in the oxidative phosphorylation pathway, as well as CASP9 being involved in the Alzheimer's disease and Parkinson's disease. Also, an additional five proteins CCL3L1, CXCL6, CCL19, CCL25, and PPBP were found to be involved in the chemokine signaling (*P* = 0.010) and cytokine–cytokine receptor interaction (*P* = 0.025) pathways. Lastly, there were five other proteins including PRB1, PRB2, PRH1, PRH2, and HTN3 that were identified to be involved in the salivary secretion pathway (*P* = 0.001).

**Table 2:** Different proteins and possible pathways related to CKD and its complications in urine samples of CKD patients compared with healthy controls.

Protein name (in alphabetical order)	Protein symbol	Mw (Da)	Theoretical Mw (Da)	CKD group (peak value)	Healthy control group (peak value)	*P*	CKD protein abundance	Pathway
Basic salivary proline-rich protein 1 or Basic salivary proline-rich protein 2	PRB1 or PRB2	5591.43	5590	0.28 (0.07–0.55)	0.19 (− 0.04 to 0.26)	0.007	High	Salivary secretion
Caspase-9	CASP9	9832.62	9828	0.20 (0.10–0.47)	0.67 (0.51–0.99)	0.000	Low	Alzheimer's disease
Cytochrome b-c1 complex subunit 8	UQCRQ	9771.01	9775	0.27 (0.13–0.57)	1.03 (0.85–1.53)	0.000	Low	Oxidative phosphorylation
								Cardiac muscle contraction
								Alzheimer's disease
Cytochrome c oxidase copper chaperone	COX17	6782.83	6784	0.24 (0.04–0.52)	0.14 (0.01–0.20)	0.045	High	Oxidative phosphorylation
Cytochrome c oxidase subunit 7B, mitochondrial	COX7B	6359.66	6362	0.29 (0.10–0.63)	0.14 (0.05–0.28)	0.020	High	Oxidative phosphorylation
								Cardiac muscle contraction
								Alzheimer's disease
C–C motif chemokine 3-like 1	CCL3L1	7796.58	7798	0.26 (0.09–0.48)	0.35 (0.24–0.56)	0.040	Low	Chemokine signaling pathway
								Cytokine–cytokine receptor interaction
C-X-C motif chemokine 6	CXCL6	8323.92	8316	0.26 (0.06–0.49)	0.08 (− 0.06 to 0.21)	0.002	High	Chemokine signaling pathway
								Cytokine–cytokine receptor interaction
C–C motif chemokine 19	CCL19	8802.89	8800	0.17 (0.03–0.35)	0.45 (0.19–0.61)	0.000	Low	Chemokine signaling pathway
								Cytokine–cytokine receptor interaction
C–C motif chemokine 25 or Cytochrome c oxidase subunit 6A2, mitochondrial	CCL25 or COX6A2	9493.79	9496	0.21 (0.12–0.41)	1.18 (0.88–1.76)	0.000	Low	Oxidative phosphorylation
								Cardiac muscle contraction
								Alzheimer's disease
Histatin-3	HTN3	1289.21	1287	1.95 (0.59–4.12)	3.69 (2.16–4.77)	0.022	Low	Salivary secretion
Platelet basic protein	PPBP	8867.85	8865	0.27 (0.11–0.55)	2.11 (1.20–3.37)	0.000	Low	Chemokine signaling pathway
								Cytokine–cytokine receptor interaction
Salivary acidic proline-rich phosphoprotein 1/2	PRH1 or PRH2	4367.30	4371	0.62 (0.08–1.18)	0.33 (0.03–0.57)	0.034	High	Salivary secretion
V-type proton ATPase subunit G 3	ATP6V1G3	14,362.9	14,363	0.10 (0.03–0.21)	0.05 (0.02–0.09)	0.005	High	Oxidative phosphorylation

The information in Table 2 shows that COX7B was upregulated and COX6A2 and UQCRQ were downregulated in the oxidative phosphorylation, cardiac muscle contraction, Alzheimer's disease, and Parkinson's disease pathways in CKD patients compared with the healthy people. In addition, ATP6V1G3 and COX17 were upregulated in the oxidative phosphorylation pathway and CASP9 was downregulated in the Alzheimer's disease and Parkinson's disease pathways in CKD patients. In the chemokine signaling and cytokine–cytokine receptor interaction pathways, CXCL6 showed greater levels while CCL3L1, CCL19, CCL25, and PPBP showed decreased levels in CKD patients than healthy controls. Additionally, PRH1, PRH2, PRB1, and PRB2 were upregulated and HTN3 was downregulated in CKD patients compared with healthy people in the salivary secretion pathway.

In order to further explore the changes of urine protein during the development of CKD, the significantly different protein peaks between CKD group and healthy control group were analyzed by comparing different stages of CKD. Because of the small sample size of CKD stage 1, we only compared the significantly different protein peaks of CKD stages 2, 3, 4, and 5. As shown in Table 3, the value of seven protein peaks changed significantly in different stages of CKD. Among these protein peaks, the related proteins associated with the two peaks (Mw = 1178.25 and Mw = 1251.78) were not identified in the UniProt database. The abundance of the peak with Mw of 1178.25 was significantly higher in CKD stage 5 than in stage 4. In addition, the abundance of the peak with Mw of 1251.78 was significantly lower in CKD stage 3 than in stage 2. The related proteins of the other five peaks were found in the UniProt database. Among them, AVP, IGHV4-31 (or TRDV2), and IGHV3-7 (or EXOC3-AS1) in CKD stage 5 were significantly downregulated compared with stage 4. The level NPB in CKD stage 4 and 5 were significantly higher compared with stage 3. Finally, HMGN4 in CKD stage 5 was significantly upregulated when compared with stage 2. However, the possible signaling pathways involved in the five proteins were not identified from the DAVID database because of small number of proteins.

**Table 3:** Significantly different protein peaks in urine samples compared between CKD stage 2, 3, 4, and 5 groups.

Mw (Da)	Theoretical Mw (Da)	CKD stage 2 group (peak value)	CKD stage 3 group (peak value)	CKD stage 4 group (peak value)	CKD stage 5 group (peak value)	*P*	Protein name	Protein symbol
1178.25	N/A	1.16 (0.70–2.37)	0.23 (− 0.13 to 1.73)	0.16 (− 0.32 to 1.35)	1.83 (0.42–3.80)^c^	0.013	N/A	N/A
1251.78	N/A	1.56 (1.11–2.46)	0.49 (− 0.47 to 1.31)^a^	0.61 (0.11–1.83)	1.29 (0.15–3.77)	0.021	N/A	N/A
3079.82	3079	0.08 (− 0.19 to 0.55)	0.02 (− 0.33 to 0.47)	0.44 (0.11–1.15)^b^	0.55 (0.09–1.09)^b^	0.008	Neuropeptide B	NPB
4017.60	4021	0.96 (0.39–1.75)	0.83 (0.29–1.50)	1.44 (0.65–2.11)	0.44 (0.07–0.89)^c^	0.013	Vasopressin-neurophysin 2-copeptin	AVP
9536.92	9539	0.11 (− 0.02 to 0.27)	0.21 (0.08–0.61)	0.24 (0.14–0.39)	0.33 (0.24–0.82)^a^	0.010	High mobility group nucleosome-binding domain-containing protein 4	HMGN4
10,782.9	10,785	0.12 (0.03–0.49)	0.16 (0.06–0.48)	0.28 (0.16–0.56)	0.14 (0.00–0.19)^c^	0.035	Immunoglobulin heavy variable 4–31 or T cell receptor delta variable 2	IGHV4–31 or TRDV2
10,853.9	10,853	0.17 (0.02–0.29)	0.20 (0.04–0.37)	0.26 (0.04–0.53)	0.05 (− 0.01 to 0.14)^c^	0.026	Immunoglobulin heavy variable 3–7 or Uncharacterized protein EXOC3-AS1	IGHV3–7 or EXOC3-AS1

^a^Means compared with CKD stage 2 group, *P* < 0.05.

^b^Means compared with CKD stage 3 group, *P* < 0.05.

^c^Means compared with CKD stage 4 group, *P* < 0.05.

## Discussion

In our research, the results showed that the chemokine signaling and cytokine–cytokine receptor interaction pathways participate in the pathogenesis of CKD. CCL3L1, CXCL6, CCL19, CCL25, and PPBP were also shown to be involved in the regulation of these pathways. CXCL6 was found to be upregulated while CCL25 was downregulated. CXCL6 has a chemotactic function for neutrophil granulocytes and CCL25 is believed to be involved in T-cell development^7,8^. Interestingly, changes in CXCL6 and CCL25 have never been mentioned in the study of CKD. We also found that CCL3L1 is downregulated. CCL3L1 has a chemotactic function for lymphocytes and monocytes^9^. Although CCL3L1 has not been found in CKD, a study has demonstrated that CCL3L1 strongly predicted the risk of lupus nephritis^10^. In other studies, CCL19 and PPBP were found to be up-regulated in CKD^11,12^. CCL19 may play a role not only in inflammatory and immunological responses but also in normal lymphocyte recirculation and homing^13^. PPBP is the chemoattractant and activator for neutrophils^14^. However, we found them to be downregulated in our study which may be due to some of the patients that we collected having undergone clinical treatment that may have affected the levels of these proteins.

CKD is associated with cardiac hypertrophy, leading to increased cardiovascular morbidity and mortality. Therefore, CKD patients have a much higher risk of cardiovascular disease than the general population. In our research, some patients also had high blood pressure and heart disease. Some researchers have found that chronic renal failure of the 5/6 nephrectomy rats were associated with mitochondrial damage^15^. Proteomic studies of rat cardiomyopathy showed that CKD cardiomyopathy was involved in mitochondrial dysfunction, oxidative phosphorylation and other pathways^16^. Mitochondria are often the primary source of reactive oxygen species (ROS), and oxidative phosphorylation is a process known to produce ROS. Damage to the mitochondrial oxidative phosphorylation pathway may lead to the leakage of electrons in the respiratory chain, impairing mitochondrial respiration and resulting in decreased mitochondrial mass and energy production while producing more ROS. Increased production of ROS can oxidize proteins, lipids and nucleic acids, thereby causing damage to cells and tissues, which may aggravate CKD and its complications^17^. Therefore, it is reasonable to infer that during the development of CKD, mitochondrial dysfunction leads to increased oxidative stress and ATP consumption in cells, which increases the production of ROS and may lead to systemic inflammation. This is one of the known pathogenic processes of CKD^18^. Scientists also have shown that there are eleven proteins involved in oxidative phosphorylation may lead to the presence of mitochondrial respiratory system damage in CKD patients. It also may be the reason for increased oxidative stress^19^. In our research, we also found some protein changes in oxidative phosphorylation pathway in patients. In this pathway, upregulation of COX7B, COX17, and ATP6V1G3 as well as downregulation of COX6A2 and UQCRQ may lead to CKD complications with cardiovascular disease. COX7B and COX6A2 are both the terminal oxidase in mitochondrial electron transport^20^. COX17 and UQCRQ are both involved in the formation of mitochondrial respiratory chain^21^. ATP6V1G3 has also been implicated in the oxidative phosphorylation pathway and has been found to be one of the novel immunohistochemical markers for chromophobe renal cell carcinoma^22^. In addition, we also found similar changes of COX7B, COX6A2 and UQCRQ also exist in the cardiac muscle contraction pathway. An abnormal cardiac muscle contraction pathway has a significant effect on coronary artery disease as well as cardiac hypertrophy and dysfunction^23,24^. The three overlapping proteins in these two pathways also provide evidence that CKD and cardiovascular disease may have some commonalities in pathogenesis.

Patients with CKD often get central nervous system disorders caused predominantly by cortical lesions or less commonly, subcortical lesions, such as cognitive decline, movement disorders, and so on^25^. Several studies have shown that when CKD occurs, the blood–brain barrier becomes more permeable, thus allowing potentially harmful substances to enter the central nervous system. The impaired blood–brain barrier in CKD patients is partially mediated by systemic inflammation, in which cytokines and chemokines can reach brain tissue and damage neurons and astrocytes^26–28^. Similarly, in our research, although only one CKD patient was also diagnosed with Alzheimer's disease, we also found proteins that are known to be components of the Alzheimer's disease and Parkinson's disease pathways in the urine of patients with CKD. This also suggests that these CKD patients may have the potential risk of Alzheimer's disease and Parkinson's disease which is in line with the findings of Korean researchers that chronic renal dysfunction may be independent risk factors for the development of Parkinson's disease in older adults^29^. In addition, proteins from the cytokine–cytokine receptor interaction and chemokine signaling pathways were also found in the urine samples of these patients. The central nervous system disorders may not only be caused directly by upregulation of COX7B and downregulation of COX6A2, UQCRQ, and CASP9, but also due to indirect regulation of CCL3L1, CCL19, CXCL6, CCL25, and PPBP (both in the cytokine–cytokine receptor interaction and chemokine signaling pathways). Among the proteins involved in the Alzheimer's disease and Parkinson's disease pathways, in addition to the fact that we already know that COX7B, COX6A2 and UQCRQ affect the function of mitochondria, downregulation of CASP9 is thought to lead to neurodegenerative diseases^30^.

Several researchers have reported a decline in the function of accessory salivary glands in patients with CKD^31^. This can lead to reduced salivary secretion and changes in salivary composition, which may cause some oral manifestations of CKD in patients such as odor of urea, dry mouth, taste alterations, pain of tongue mucosa, periodontitis, high prevalence of dental calculus and more^32^. Similar phenomena were observed on studies with Wistar rats^33^. Our results showed that upregulation of PRB1, PRB2, PRH1, and PRH2 as well as downregulation of HTN3 in the salivary secretion pathway of CKD patients. PRB1 and PRB2 are involved in the production of proline in saliva^34^. PRH1 and PRH2 can inhibit the growth of calcium phosphate crystal, and upregulation of the proteins will make dental enamel lose the protective and reparative environment^35^. HTN3 is involved in the formation of histatin in saliva which not only participates in the formation of enamel pellicle, but also has antibacterial and antifungal activities^36,37^. Therefore, the changes of these proteins affect the levels of proline, histatin, and calcium phosphate crystal contents in oral cavity which may cause a variety of oral diseases.

In addition, we found that the levels of five proteins also changed in different stages of CKD. Compared with the healthy control group, proteins NPB, AVP, IGHV4-31 (or TRDV2), and IGHV3-7 (or EXOC3-AS1) expression in CKD group were upregulated. In CKD stages 2, 3, 4, and 5, NPB was upregulated most in stages 4 and 5, which was significantly upregulated when compared with stage 3. NPB is ubiquitously expressed in the central nervous system^38^. Its high expression in the middle and late stage of CKD may be associated with the central nervous system disorders. AVP, IGHV4-31 (TRDV2), and IGHV3-7 (EXOC3-AS1) were upregulated most in stage 4, which was significantly upregulated compared with stage 5. AVP has previously been shown to affect the urination of the kidney^39^. We found that protein AVP was continuously overexpressed in CKD stages 2, 3, and 4, peaked in stage 4 and decreased in stage 5, which may be related to the symptoms of decreased urine output at the end of CKD. Because of the same Mw value, proteins IGHV4-31, IGHV3-7, TRDV2, and EXOC3-AS1 may appear. The upregulation of IGHV and TRDV2 may affect the changes of antibodies and T cells. The antibodies and immune cells can lead to chronic renal injury^40^. It is interesting that the abundances of proteins associated with IGHV and TRDV2 reached the highest value in CKD stage 4 and then decrease in stage 5; however the mechanism needs to be further studied. The function of EXOC3-AS1 has not yet been discovered. HMGN4 level in the CKD group were downregulated compared with the healthy control group and the protein was down-regulated most in stages 2, which was significantly downregulated compared with stage 5. HMGN4 is a kind of nuclear protein whose function has not been fully elucidated^41^. Previous studies have only shown that the upregulation of HMGN4 was related to the occurrence of thyroid cancer and hepatocellular carcinoma^42,43^. The role of the protein in the development of CKD has not been reported. In this study, because the cases number of CKD stage 1 was small, we did not analyze stage 1 with other stages. Therefore, we will expand the sample size and improve the current results in future studies.

**Figure 2:**
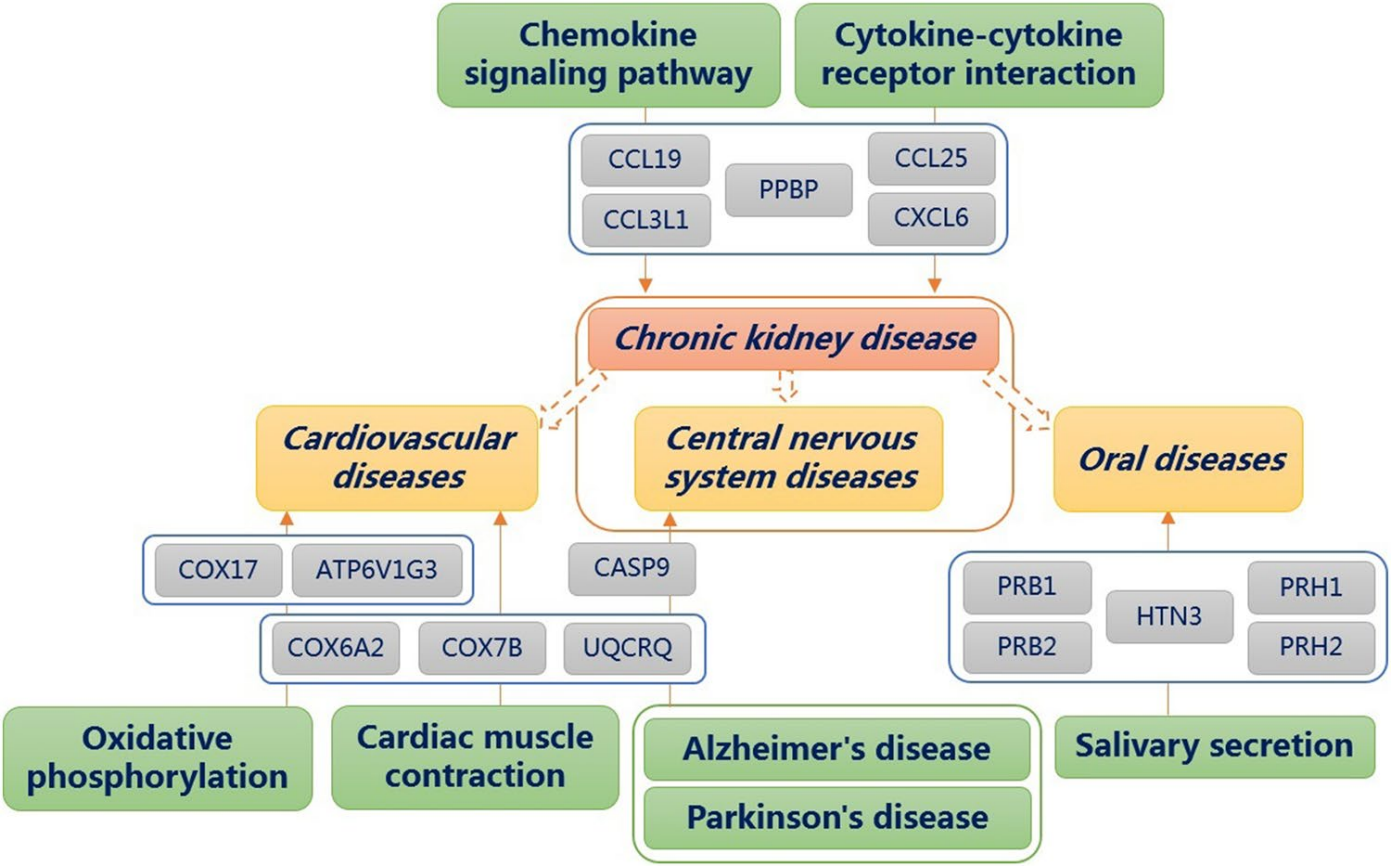
The possible pathways involved in the progression of CKD. Cardiovascular diseases, central nervous system diseases, and oral diseases are complications of CKD. Regulation by CCL3L1, CCL19, CXCL6, CCL25, and PPBP (chemokine signaling pathway and cytokine–cytokine receptor interaction) may affect the progress of CKD and central nervous system diseases. The central nervous system diseases may also be regulated by COX6A2, COX7B, UQCRQ, and CASP9 (Alzheimer's disease and Parkinson's disease). COX6A2, COX7B, and UQCRQ may also cause cardiovascular diseases by regulating pathway of oxidative phosphorylation and cardiac muscle contraction. Cardiovascular diseases may also be regulated by COX17 and ATP6V1G3 (oxidative phosphorylation). In addition, oral diseases may be regulated by HTN3, PRB1, PRB2, PRH1, and PRH2 (salivary secretion).

In summary, several pathways that we found that have changed are consistent with the occurrence and development of CKD in clinic. We constructed a schematic diagram of possible pathways involved in the progression of CKD (Fig. 2). Although some of these pathways such as the chemokine signaling, cytokine–cytokine receptor interaction, and oxidative phosphorylation pathways have been detected in the serum sample of CKD patients, they are rarely mentioned in urine samples test. Consequently, the protein regulations involved in these pathways found in our work have not been reported before. The results will need to be further studied to verify the initial findings. However, these proteins may provide a new research interest about the pathogenesis of CKD. In addition, we also found that there were five proteins with significantly different abundances in CKD stages 2, 3, 4, and 5. As some novel biomarkers, these regulated proteins may be used for noninvasive and convenient diagnosis of CKD and its complications through urine testing in the future.

## Supplementary information

Supplementary Tables.
