# Supplementary material for: Changes of protein levels in human urine reflect the dysregulation of signaling pathways of chronic kidney disease and its complications
Source: Sci Rep. 2020 Nov 27;10:20743. doi: 10.1038/s41598-020-77916-z (PMC7699629; doi:10.1038/s41598-020-77916-z)
Supplement: Supplementary file 1 — Supplementary Tables. [file 41598_2020_77916_MOESM1_ESM.docx]

**Supplementary**

**Table S1. All significantly different protein peaks in urine samples of CKD compared with healthy controls**

| **Mw (Da)** | **CKD group (peak value)** | **Healthy control group (peak value)** | ***P*** |
| --- | --- | --- | --- |
| 1001.20 | 7.12(2.09-15.35) | 16.07(6.48-20.52) | 0.002073 |
| 1015.61 | 4.95(1.79-11.88) | 10.22(4.72-15.91) | 0.003765 |
| 1017.19 | 5.61(2.02-13.10) | 15.14(7.39-21.02) | 0.000147 |
| 1018.38 | 4.81(1.90-12.19) | 13.03(6.96-19.41) | 0.000297 |
| 1020.07 | 3.84(1.42-8.61) | 6.72(4.34-13.97) | 0.015274 |
| 1026.86 | 2.70(0.68-7.28) | 6.21(3.62-10.49) | 0.009839 |
| 1027.83 | 2.86(0.63-7.56) | 5.58(3.63-12.26) | 0.006115 |
| 1028.85 | 3.34(1.33-9.48) | 6.67(4.64-11.34) | 0.011181 |
| 1030.85 | 3.88(1.65-10.17) | 7.37(3.62-12.26) | 0.018319 |
| 1033.82 | 2.80(0.73-10.83) | 9.21(7.19-13.84) | 0.000793 |
| 1042.03 | 2.17(0.87-5.57) | 5.56(1.97-8.85) | 0.034093 |
| 1042.97 | 2.19(0.69-6.46) | 7.30(2.78-10.92) | 0.003312 |
| 1043.94 | 3.40(1.01-8.99) | 8.44(5.36-13.43) | 0.001107 |
| 1045.31 | 2.98(0.95-9.03) | 8.22(3.34-12.03) | 0.004098 |
| 1056.94 | 4.54(0.99-11.51) | 10.06(6.68-13.67) | 0.001784 |
| 1071.64 | 3.28(0.27-11.69) | 11.84(8.34-18.47) | 2.94E-05 |
| 1072.22 | 3.22(0.09-12.94) | 13.67(10.91-22.29) | 7.15E-06 |
| 1073.80 | 4.79(0.85-15.46) | 16.70(13.18-24.58) | 5.33E-06 |
| 1085.53 | 1.69(0.00-6.81) | 6.35(2.98-10.49) | 0.010357 |
| 1086.61 | 2.37(0.17-7.64) | 9.66(4.62-15.74) | 0.003457 |
| 1089.15 | 2.79(0.50-10.46) | 10.31(6.86-18.82) | 0.000104 |
| 1096.30 | 1.76(0.01-6.44) | 5.13(2.68-11.38) | 0.014363 |
| 1097.34 | 2.21(0.29-6.31) | 6.56(3.51-11.01) | 0.000536 |
| 1098.30 | 2.12(0.12-7.50) | 8.91(6.52-13.23) | 3.79E-05 |
| 1099.01 | 3.94(0.26-10.75) | 12.96(9.35-17.45) | 4.31E-06 |
| 1101.95 | 5.27(0.29-12.21) | 14.05(10.80-21.24) | 2.15E-05 |
| 1111.66 | 1.99(0.30-4.97) | 5.02(1.91-8.41) | 0.008418 |
| 1114.61 | 3.74(0.52-11.10) | 16.58(11.52-23.70) | 1.80E-07 |
| 1118.04 | 8.12(1.31-22.39) | 33.45(25.62-43.05) | 1.52E-08 |
| 1121.47 | 1.52(0.35-5.06) | 6.61(3.08-9.32) | 0.000536 |
| 1130.35 | 1.00(-0.20-4.27) | 3.82(1.76-6.47) | 0.000308 |
| 1131.46 | 1.90(0.02-6.73) | 8.64(4.30-13.04) | 5.33E-06 |
| 1134.16 | 2.96(1.13-8.60) | 12.29(8.14-16.51) | 4.04E-07 |
| 1137.01 | 2.29(0.65-5.57) | 4.14(2.17-6.99) | 0.023197 |
| 1138.48 | 2.53(0.24-5.99) | 4.62(2.57-6.97) | 0.016838 |
| 1143.79 | 1.06(0.15-2.90) | -0.05(-0.74-0.94) | 0.011324 |
| 1148.03 | -0.08(-0.90-1.43) | -1.70(-2.77--0.23) | 6.83E-05 |
| 1150.51 | -0.07(-1.22-1.44) | -1.63(-3.05--0.65) | 9.36E-06 |
| 1155.49 | 1.31(0.06-3.08) | 3.27(1.75-5.85) | 0.000411 |
| 1163.00 | 0.84(-0.15-2.59) | -0.10(-1.82-1.10) | 0.001533 |
| 1166.61 | 0.01(-1.14-2.20) | -1.40(-2.46--0.34) | 0.000454 |
| 1174.91 | 0.49(-0.24-2.52) | 2.65(1.00-4.10) | 0.001314 |
| 1178.25 | 0.82(-0.07-2.60) | 3.67(1.08-7.97) | 0.001784 |
| 1186.80 | 0.89(0.05-2.21) | 0.02(-1.08-1.41) | 0.016838 |
| 1194.05 | 0.71(-0.05-2.52) | 3.57(0.92-6.97) | 0.000125 |
| 1196.57 | 0.76(-0.29-2.12) | 2.44(0.59-5.82) | 0.000859 |
| 1199.14 | 1.97(0.36-4.56) | 8.01(2.26-9.81) | 1.56E-05 |
| 1201.68 | 1.24(0.12-2.92) | 2.70(1.23-3.88) | 0.038447 |
| 1213.38 | 0.46(-0.39-2.37) | 2.02(0.47-5.91) | 0.005871 |
| 1216.67 | 0.83(-0.10-3.02) | 3.15(0.95-6.37) | 0.001629 |
| 1218.44 | 1.17(0.29-2.56) | 2.46(1.54-5.10) | 0.001580 |
| 1219.07 | 1.84(0.41-3.56) | 2.68(1.86-6.59) | 0.002627 |
| 1222.13 | 2.03(0.41-4.74) | 5.32(1.45-7.81) | 0.014904 |
| 1236.51 | 1.47(0.40-3.98) | 4.99(1.65-6.19) | 0.003507 |
| 1244.05 | 1.67(0.35-3.71) | 3.21(1.11-7.53) | 0.009223 |
| 1248.29 | 2.15(0.50-4.45) | 3.55(1.62-5.85)) | 0.043265 |
| 1249.51 | 1.73(0.32-3.51) | 3.37(2.09-6.01) | 0.002043 |
| 1250.61 | 1.64(0.61-3.28) | 2.91(1.30-4.80) | 0.020151 |
| 1251.78 | 1.17(0.30-3.21) | 2.61(1.27-5.15) | 0.020633 |
| 1258.09 | 1.22(0.37-3.13) | 2.86(1.30-5.64) | 0.014904 |
| 1260.64 | 1.13(0.22-3.14) | 3.11(1.56-4.05) | 0.003082 |
| 1262.14 | 1.39(0.37-3.41) | 3.12(1.64-5.30) | 0.002267 |
| 1263.97 | 1.38(0.21-3.79) | 4.24(2.30-5.73) | 0.000411 |
| 1269.26 | 2.17(0.51-4.27) | 0.91(-0.12-2.65) | 0.034471 |
| 1273.79 | 1.83(0.56-4.09) | 3.94(2.67-5.48) | 0.001867 |
| 1276.75 | 2.14(0.71-5.49) | 5.00(2.65-6.52) | 0.001953 |
| 1279.42 | 1.89(0.40-4.30) | 3.57(1.81-5.72) | 0.027877 |
| 1289.21 | 1.95(0.59-4.12) | 3.69(2.16-4.77) | 0.021627 |
| 1292.31 | 3.18(1.54-6.47) | 8.09(6.05-11.96) | 2.47E-05 |
| 1306.26 | 2.07(0.82-4.99) | 4.57(2.43-7.43) | 0.003218 |
| 1310.00 | 1.03(0.00-3.27) | 3.74(1.93-5.14) | 0.004458 |
| 1317.63 | 1.94(0.47-4.23) | 3.48(2.12-5.17) | 0.022140 |
| 1321.01 | 2.01(0.41-5.05) | 5.84(3.57-9.28) | 8.22E-05 |
| 1334.04 | 1.95(0.24-4.83) | 7.69(4.53-10.91) | 7.70E-07 |
| 1336.17 | 2.55(0.44-7.07) | 12.38(8.46-17.44) | 3.47E-09 |
| 1338.89 | 1.90(0.48-5.52) | 6.55(4.36-9.86) | 6.86E-06 |
| 1351.40 | 1.18(0.08-3.73) | 4.05(1.69-5.80) | 0.000708 |
| 1353.83 | 0.83(0.00-2.94) | 3.70(1.35-6.21) | 0.002136 |
| 1361.86 | 0.68(-0.15-1.93) | -0.78(-1.20--0.07) | 1.53E-05 |
| 1384.46 | 0.41(-0.40-1.64) | -0.58(-1.26-0.04) | 0.000158 |
| 1397.43 | 1.06(0.00-2.56) | 2.53(1.62-5.89) | 0.001274 |
| 1399.80 | 0.36(-0.16-1.39) | 1.57(-0.03-3.37) | 0.031901 |
| 1401.71 | 0.53(-0.35-1.99) | 1.32(0.45-3.78) | 0.020878 |
| 1411.72 | 0.56(0.01-1.87) | 1.49(0.55-2.97) | 0.007677 |
| 1415.13 | 0.55(-0.09-1.87) | 2.09(0.75-4.85) | 0.003265 |
| 1419.45 | 1.16(0.03-3.59) | 4.00(1.32-8.41) | 0.000632 |
| 1421.46 | 1.21(-0.03-2.48) | 2.19(0.94-5.76) | 0.003819 |
| 1424.63 | 1.05(-0.03-2.78) | 2.73(1.48-5.49) | 0.001604 |
| 1434.54 | 1.23(0.12-2.77) | 4.26(1.62-8.52) | 8.27E-06 |
| 1436.57 | 1.22(0.10-2.67) | 3.27(1.10-6.92) | 0.001895 |
| 1438.87 | 1.21(0.05-2.82) | 4.35(1.61-8.16) | 2.94E-05 |
| 1441.73 | 0.80(0.08-2.67) | 3.93(1.65-7.84) | 2.56E-05 |
| 1443.72 | 1.13(0.29-2.85) | 2.58(1.06-5.92) | 0.003457 |
| 1444.54 | 1.45(0.07-3.33) | 3.02(0.82-6.11) | 0.003038 |
| 1446.78 | 2.02(0.66-4.86) | 4.00(1.61-8.49) | 0.017671 |
| 1451.39 | 1.38(0.24-3.56) | 3.79(1.19-8.14) | 0.001441 |
| 1454.56 | 1.58(0.46-3.69) | 3.46(1.10-5.77) | 0.006115 |
| 1456.16 | 1.70(0.55-3.66) | 3.16(1.52-8.74) | 0.005410 |
| 1457.73 | 1.37(0.41-2.98) | 3.53(1.25-7.68) | 0.001705 |
| 1460.16 | 1.82(0.52-3.70) | 3.41(1.92-5.20) | 0.011181 |
| 1462.34 | 2.07(0.69-4.48) | 7.47(2.85-11.50) | 7.49E-05 |
| 1464.74 | 1.88(0.39-3.97) | 4.93(1.56-8.09) | 0.000103 |
| 1467.81 | 2.55(0.90-4.92) | 4.38(2.59-9.42) | 0.001464 |
| 1471.01 | 2.26(0.79-4.14) | 4.16(1.93-7.64) | 0.004458 |
| 1479.06 | 2.71(0.94-5.47) | 5.11(2.12-9.65) | 0.002867 |
| 1483.77 | 2.73(0.77-5.50) | 6.18(2.55-8.46) | 0.000756 |
| 1485.98 | 1.32(0.43-3.53) | 3.58(1.96-8.19) | 0.001533 |
| 1487.62 | 1.86(0.54-3.84) | 3.90(1.53-6.07) | 0.002588 |
| 1490.62 | 2.00(0.74-4.07) | 3.28(2.09-7.12) | 0.009223 |
| 1493.64 | 2.07(0.74-4.34) | 5.14(2.56-7.75) | 0.000173 |
| 1495.74 | 2.05(0.59-4.19) | 4.94(2.85-7.07) | 0.000161 |
| 1498.56 | 2.37(0.77-4.74) | 4.94(1.89-7.21) | 0.003765 |
| 1501.44 | 2.25(0.85-4.84) | 4.00(1.66-6.16) | 0.026942 |
| 1507.83 | 2.36(1.13-4.72) | 4.20(2.43-6.69) | 0.016236 |
| 1512.03 | 2.70(1.14-5.03) | 5.71(4.58-7.82) | 3.79E-05 |
| 1524.33 | 1.89(0.66-4.15) | 3.29(1.83-5.00) | 0.028839 |
| 1529.83 | 2.07(0.74-3.84) | 3.44(1.81-4.69) | 0.012216 |
| 1540.49 | 1.65(0.53-3.30) | 4.07(2.70-5.02) | 0.000259 |
| 1552.03 | 1.17(0.21-2.48) | 1.83(1.47-3.36) | 0.003127 |
| 1554.82 | 1.62(0.34-3.47) | 4.18(3.41-5.63) | 2.51E-06 |
| 1557.33 | 1.12(0.36-2.47) | 3.00(2.02-4.26) | 4.04E-06 |
| 1581.88 | 0.22(-0.34-1.57) | -0.29(-1.00--0.01) | 0.000221 |
| 1588.18 | 0.29(-0.54-1.72) | -0.17(-0.75-0.26) | 0.008200 |
| 1594.46 | 0.45(-0.08-2.00) | 0.18(-0.17-0.51) | 0.040144 |
| 1599.04 | 0.44(-0.23-1.56) | -0.01(-0.70-0.50) | 0.009343 |
| 1637.59 | 0.42(-0.17-1.40) | 1.60(0.33-2.17) | 0.008529 |
| 1669.66 | 0.54(-0.02-1.84) | 1.55(0.79-3.27) | 0.010357 |
| 1684.76 | 1.14(0.17-2.25) | 3.19(0.82-3.91) | 0.001679 |
| 1689.67 | 1.11(0.24-2.61) | 2.55(1.20-3.81) | 0.008986 |
| 1695.95 | 1.05(0.15-2.20) | 1.83(0.89-2.95) | 0.008641 |
| 1699.99 | 0.78(0.14-1.92) | 2.01(0.70-4.72) | 0.004520 |
| 1702.01 | 1.26(0.34-2.36) | 2.03(1.29-4.25) | 0.004274 |
| 1704.33 | 1.31(0.25-2.39) | 1.91(1.10-4.95) | 0.017885 |
| 1707.96 | 1.20(0.28-2.15) | 1.74(0.94-4.01) | 0.014904 |
| 1711.57 | 0.83(0.23-2.26) | 2.13(1.16-3.75) | 0.001216 |
| 1720.16 | 1.13(0.26-2.64) | 2.46(1.27-3.62) | 0.002909 |
| 1724.68 | 1.20(0.12-2.56) | 1.99(1.24-3.77) | 0.037215 |
| 1729.98 | 1.00(0.06-2.64) | 2.94(1.79-4.58) | 1.04E-05 |
| 1731.72 | 1.00(0.23-2.43) | 2.30(1.69-4.77) | 0.000114 |
| 1752.75 | 0.86(0.15-2.06) | 1.72(0.71-2.97) | 0.016236 |
| 1800.13 | 1.02(0.18-2.18) | 0.19(-0.26-0.47) | 0.000384 |
| 1805.50 | 0.36(-0.09-1.58) | 0.01(-0.47-0.33) | 0.008418 |
| 1809.65 | 0.57(-0.08-2.01) | -0.12(-0.37-0.53) | 0.003765 |
| 1814.91 | 0.44(0.01-1.47) | 0.11(-0.29-0.57) | 0.015274 |
| 1831.93 | 0.29(-0.17-1.36) | -0.07(-0.32-0.30) | 0.006198 |
| 1837.34 | 0.43(0.00-1.56) | 0.18(-0.37-0.44) | 0.007576 |
| 1841.46 | 0.36(-0.20-1.58) | 0.07(-0.32-0.38) | 0.033719 |
| 1911.54 | 0.28(-0.13-1.65) | 1.13(0.40-2.13) | 0.020390 |
| 1932.75 | 0.65(0.01-2.02) | 1.34(0.64-2.70) | 0.026034 |
| 1938.35 | 0.69(0.11-1.88) | 1.80(0.78-3.89) | 0.002335 |
| 1941.26 | 0.61(0.16-1.83) | 0.94(0.62-2.98) | 0.019446 |
| 1956.01 | 0.58(0.02-1.74) | 1.37(0.55-2.66) | 0.009966 |
| 1961.08 | 1.28(0.29-2.66) | 2.31(0.90-3.75) | 0.034471 |
| 1976.97 | 1.07(0.21-2.58) | 2.03(0.76-3.16) | 0.014363 |
| 2017.85 | 1.08(0.31-2.45) | 0.54(0.03-1.19) | 0.043728 |
| 2022.01 | 1.18(0.15-2.17) | 0.56(-0.26-0.93) | 0.006902 |
| 2024.91 | 0.86(0.24-1.88) | 0.43(0.00-1.12) | 0.027562 |
| 2037.83 | 1.22(0.16-2.35) | 0.49(0.14-1.17) | 0.042807 |
| 2042.54 | 0.65(0.04-1.98) | 0.16(-0.21-0.69) | 0.009966 |
| 2052.42 | 0.94(0.17-2.07) | -0.02(-0.26-0.24) | 4.22E-06 |
| 2055.81 | 0.74(0.11-1.82) | 0.14(-0.16-0.48) | 0.004156 |
| 2065.54 | 0.41(-0.2-1.50) | 0.09(-0.36-0.63) | 0.045619 |
| 2151.77 | 0.72(-0.01-1.97) | 1.79(0.93-2.42) | 0.001274 |
| 2168.76 | 0.91(0.05-2.23) | 2.05(1.15-2.78) | 0.002588 |
| 2255.48 | 0.79(0.16-1.79) | 0.37(0.06-0.76) | 0.022929 |
| 2262.13 | 0.82(0.32-1.88) | 0.38(-0.16-1.04) | 0.029497 |
| 2279.98 | 0.58(-0.08-1.55) | 0.06(-0.32-0.33) | 0.009966 |
| 2305.78 | 0.63(0.03-1.31) | 0.21(-0.15-0.61) | 0.008200 |
| 2341.91 | 0.57(0.01-1.81) | 0.25(-0.11-0.46) | 0.020633 |
| 2365.91 | 0.49(-0.08-1.61) | 0.00(-0.34-0.35) | 0.005263 |
| 2527.46 | 0.80(0.14-2.27) | 0.00(-0.29-0.30) | 3.64E-05 |
| 2539.20 | 0.40(0.01-1.41) | 0.13(-0.39-0.46) | 0.008200 |
| 2549.39 | 0.75(0.12-1.91) | 0.11(-0.32-0.56) | 0.000308 |
| 2628.18 | 0.56(0.05-1.59) | 0.27(-0.04-0.55) | 0.026034 |
| 2662.54 | 0.64(0.02-1.61) | 0.27(-0.04-0.71) | 0.046590 |
| 2737.31 | 1.15(0.24-2.66) | 4.33(1.89-10.35) | 1.13E-06 |
| 2864.12 | 0.40(-0.10-1.00) | 0.05(-0.22-0.44) | 0.040144 |
| 2878.04 | 0.60(0.07-1.36) | 0.19(-0.10-0.66) | 0.020151 |
| 2932.43 | 0.49(0.00-1.25) | 1.81(1.05-3.50) | 9.96E-06 |
| 3055.01 | 0.35(-0.04-1.08) | 0.10(-0.32-0.47) | 0.018763 |
| 3079.82 | 0.41(-0.07-1.13) | -0.06(-0.23-0.21) | 0.001839 |
| 3297.52 | 0.77(0.20-1.66) | 1.43(0.65-4.87) | 0.005263 |
| 3728.72 | 0.56(0.06-1.33) | 0.17(-0.05-0.36) | 0.008986 |
| 3822.20 | 0.54(0.08-1.32) | 0.09(-0.18-0.52) | 0.002301 |
| 3988.30 | 0.56(0.14-1.20) | 0.25(0.04-0.44) | 0.028195 |
| 4017.60 | 0.59(0.13-1.51) | 0.20(0.00-0.47) | 0.002951 |
| 4025.78 | 0.47(0.08-0.99) | -0.06(-0.28-0.42) | 0.000390 |
| 4038.06 | 0.74(0.16-1.42) | 0.36(0.01-0.87) | 0.034852 |
| 4054.55 | 0.87(0.24-1.30) | 0.19(-0.12-0.65) | 0.000447 |
| 4076.54 | 0.64(0.23-1.33) | 0.38(0.17-0.72) | 0.039288 |
| 4107.26 | 0.71(0.30-1.18) | 0.35(-0.09-0.58) | 0.000793 |
| 4162.73 | 0.77(0.26-1.51) | 0.41(0.14-0.75) | 0.014363 |
| 4184.52 | 0.74(0.20-1.41) | 0.21(-0.16-0.49) | 0.000359 |
| 4206.29 | 0.65(0.12-1.34) | 0.22(0.03-0.50) | 0.001629 |
| 4246.28 | 0.81(0.17-1.67) | 0.33(0.16-0.50) | 0.003608 |
| 4262.02 | 0.98(0.25-2.40) | 0.46(0.27-1.01) | 0.018101 |
| 4278.20 | 0.84(0.27-1.71) | 0.30(-0.01-0.86) | 0.001090 |
| 4300.03 | 0.70(0.18-1.28) | 0.41(0.14-0.70) | 0.036413 |
| 4324.48 | 0.83(0.33-1.69) | 0.41(0.16-0.71) | 0.004156 |
| 4340.31 | 0.75(0.11-1.39) | 0.14(-0.08-0.52) | 0.000377 |
| 4367.30 | 0.62(0.08-1.18) | 0.33(0.03-0.57) | 0.033719 |
| 4426.51 | 0.58(0.16-1.19) | 0.26(0.04-0.35) | 0.005336 |
| 4573.83 | 0.50(0.12-1.08) | 0.23(-0.13-0.49) | 0.005484 |
| 4649.81 | 0.74(0.21-1.23) | 5.83(3.86-9.07) | 1.67E-12 |
| 4756.84 | 0.71(0.26-1.72) | 0.45(0.20-0.74) | 0.028515 |
| 5553.90 | 0.26(-0.02-0.57) | -0.02(-0.11-0.18) | 0.001705 |
| 5591.43 | 0.28(0.07-0.55) | 0.19(-0.04-0.26) | 0.006720 |
| 5744.52 | 0.67(0.17-1.38) | 0.32(0.01-0.41) | 0.000153 |
| 5876.83 | 0.38(0.15-0.80) | 0.11(-0.04-0.30) | 0.000371 |
| 5922.58 | 0.40(0.16-0.75) | 0.22(0.07-0.45) | 0.035625 |
| 6043.83 | 0.44(0.14-0.81) | 1.81(1.10-3.22) | 4.92E-09 |
| 6199.08 | 0.50(0.24-1.01) | 0.90(0.50-1.54) | 0.021882 |
| 6359.66 | 0.29(0.10-0.63) | 0.14(0.05-0.28) | 0.019678 |
| 6447.81 | 0.25(0.04-0.52) | 0.06(-0.09-0.25) | 0.006902 |
| 6782.83 | 0.24(0.04-0.52) | 0.14(0.01-0.20) | 0.044665 |
| 6944.25 | 0.22(0.06-0.55) | 0.01(-0.08-0.18) | 0.000341 |
| 7204.07 | 0.28(0.10-0.48) | 0.09(-0.08-0.25) | 0.001142 |
| 7315.10 | 0.30(0.12-0.50) | 0.14(0.02-0.24) | 0.001811 |
| 7796.58 | 0.26(0.09-0.48) | 0.35(0.24-0.56) | 0.039714 |
| 7834.01 | 0.40(0.15-0.74) | 1.44(1.13-1.71) | 1.50E-10 |
| 8323.92 | 0.26(0.06-0.49) | 0.08(-0.06-0.21) | 0.001604 |
| 8374.59 | 0.25(0.09-0.48) | 0.08(-0.07-0.20) | 0.000756 |
| 8504.70 | 0.17(0.05-0.33) | 0.08(-0.01-0.13) | 0.003712 |
| 8654.96 | 0.33(0.13-0.58) | 0.49(0.31-0.67) | 0.026942 |
| 8802.89 | 0.17(0.03-0.35) | 0.45(0.19-0.61) | 0.000264 |
| 8867.85 | 0.27(0.11-0.55) | 2.11(1.20-3.37) | 6.99E-13 |
| 9105.42 | 0.16(0.03-0.35) | 0.44(0.24-0.68) | 0.000150 |
| 9493.79 | 0.21(0.12-0.41) | 1.18(0.88-1.76) | 8.86E-12 |
| 9536.92 | 0.24(0.10-0.54) | 4.91(2.92-6.65) | 1.75E-13 |
| 9635.81 | 0.25(0.10-0.49) | 1.53(1.12-2.49) | 1.45E-11 |
| 9696.17 | 0.24(0.12-0.50) | 1.42(1.00-1.95) | 1.68E-10 |
| 9771.01 | 0.27(0.13-0.57) | 1.03(0.85-1.53) | 9.67E-07 |
| 9832.62 | 0.20(0.10-0.47) | 0.67(0.51-0.99) | 1.53E-05 |
| 10782.9 | 0.17(0.07-0.39) | 0.33(0.17-0.42) | 0.006033 |
| 10853.9 | 0.18(0.04-0.36) | 0.06(0.01-0.11) | 0.000664 |
| 10968.2 | 0.17(0.04-0.42) | 0.06(0.03-0.12) | 0.002665 |
| 11660.4 | 0.62(0.21-1.50) | 0.23(0.13-0.31) | 3.18E-05 |
| 11742.4 | 0.71(0.31-2.03) | 0.12(0.08-0.23) | 3.56E-09 |
| 11904.6 | 0.40(0.21-1.01) | 0.13(0.03-0.19) | 5.99E-07 |
| 13297.8 | 0.12(0.03-0.22) | 0.05(0.01-0.08) | 0.003127 |
| 13361.8 | 0.11(0.02-0.23) | 0.06(0.01-0.09) | 0.006033 |
| 13586.4 | 0.18(0.06-0.37) | 0.07(0.04-0.09) | 0.000225 |
| 13892.0 | 0.11(0.04-0.22) | 0.04(0.03-0.09) | 0.004845 |
| 14362.9 | 0.10(0.03-0.21) | 0.05(0.02-0.09) | 0.005191 |
| 15483.2 | 0.14(0.06-0.21) | 0.07(0.03-0.11) | 0.024580 |
| 15695.9 | 0.08(0.04-0.17) | 0.04(0.00-0.10) | 0.018988 |
| 15909.1 | 0.11(0.03-0.20) | 0.03(0.02-0.06) | 0.000510 |

**Table S2.** **Significantly different protein peaks** **identified in urine samples of CKD compared with healthy controls**

| **Mw (Da)** | **Theoretical Mw (Da)** | **CKD group**  **(peak value)** | **Healthy control group**  **(peak value)** | ***P*** | **Protein** **name** | **Protein symbol** | **CKD protein abundance** |
| --- | --- | --- | --- | --- | --- | --- | --- |
| 1018.38 | 1019 | 4.81(1.90-12.19) | 13.03(6.96-19.41) | 0.000297 | Urotensin-2B | UTS2B | low |
| 1042.97 | 1043 | 2.19(0.69-6.46) | 7.30(2.78-10.92) | 0.003312 | Rho GTPase-activating protein 45 | ARHGAP45 | low |
| 1045.31 | 1046 | 2.98(0.95-9.03) | 8.22(3.34-12.03) | 0.004098 | Angiotensinogen | AGT | low |
| 1085.53 | 1085 | 1.69(0.00-6.81) | 6.35(2.98-10.49) | 0.010357 | Tachykinin-4 | TAC4 | low |
| 1086.61 | 1087 | 2.37(0.17-7.64) | 9.66(4.62-15.74) | 0.003457 | Vasopressin-neurophysin 2-copeptin | AVP | low |
| 1099.01 | 1100 | 3.94(0.26-10.75) | 12.96(9.35-17.45) | 4.31E-06 | Proenkephalin-B | PDYN | low |
| 1121.47 | 1121 | 1.52(0.35-5.06) | 6.61(3.08-9.32) | 0.000536 | Gastrin-releasing peptide | GRP | low |
| 1134.16 | 1134 | 2.96(1.13-8.60) | 12.29(8.14-16.51) | 4.04E-07 | Protachykinin-1 | TAC1 | low |
| 1163.00 | 1162 | 0.84(-0.15-2.59) | -0.10(-1.82-1.10) | 0.001533 | Chromogranin-A | CHGA | high |
| 1199.14 | 1200 | 1.97(0.36-4.56) | 8.01(2.26-9.81) | 1.56E-05 | Progonadoliberin-1 | GNRH1 | low |
| 1262.14 | 1263 | 1.39(0.37-3.41) | 3.12(1.64-5.30) | 0.002267 | Chymotrypsinogen B or Chymotrypsinogen B2 | CTRB1 or CTRB2 | low |
| 1289.21 | 1287 | 1.95(0.59-4.12) | 3.69(2.16-4.77) | 0.021627 | Histatin-3 | HTN3 | low |
| 1310.00 | 1309 | 1.03(0.00-3.27) | 3.74(1.93-5.14) | 0.004458 | Hemoglobin subunit beta | HBB | low |
| 1336.17 | 1335 | 2.55(0.44-7.07) | 12.38(8.46-17.44) | 3.47E-09 | Histatin-3 | HTN3 | low |
| 1399.80 | 1399 | 0.36(-0.16-1.39) | 1.57(-0.03-3.37) | 0.031901 | Minor histocompatibility protein HMSD variant form | HMSD | low |
| 1411.72 | 1411 | 0.56(0.01-1.87) | 1.49(0.55-2.97) | 0.007677 | Podoplanin | PDPN | low |
| 1434.54 | 1435 | 1.23(0.12-2.77) | 4.26(1.62-8.52) | 8.27E-06 | Submaxillary gland androgen-regulated protein 3B or Histatin-3 | SMR3B or HTN3 | low |
| 1443.72 | 1444 | 1.13(0.29-2.85) | 2.58(1.06-5.92) | 0.003457 | Histatin-3 | HTN3 | low |
| 1446.78 | 1446 | 2.02(0.66-4.86) | 4.00(1.61-8.49) | 0.017671 | Vitamin K-dependent protein C | PROC | low |
| 1451.39 | 1449 | 1.38(0.24-3.56) | 3.79(1.19-8.14) | 0.001441 | Pro-MCH | PMCH | low |
| 1464.74 | 1463 | 1.88(0.39-3.97) | 4.93(1.56-8.09) | 0.000103 | Guanylin | GUCA2A | low |
| 1490.62 | 1492 | 2.00(0.74-4.07) | 3.28(2.09-7.12) | 0.009223 | Histatin-3 | HTN3 | low |
| 1501.44 | 1502 | 2.25(0.85-4.84) | 4.00(1.66-6.16) | 0.026942 | Neuroendocrine protein 7B2 | SCG5 | low |
| 1552.03 | 1551 | 1.17(0.21-2.48) | 1.83(1.47-3.36) | 0.003127 | Apelin | APLN | low |
| 1689.67 | 1690 | 1.11(0.24-2.61) | 2.55(1.20-3.81) | 0.008986 | Neurotensin/neuromedin N | NTS | low |
| 1699.99 | 1699 | 0.78(0.14-1.92) | 2.01(0.70-4.72) | 0.00452 | Tryptase gamma | TPSG1 | low |
| 1720.16 | 1719 | 1.13(0.26-2.64) | 2.46(1.27-3.62) | 0.002909 | Histatin-3 | HTN3 | low |
| 1800.13 | 1798 | 1.02(0.18-2.18) | 0.19(-0.26-0.47) | 0.000384 | ProSAAS | PCSK1N | high |
| 1809.65 | 1809 | 0.57(-0.08-2.01) | -0.12(-0.37-0.53) | 0.003765 | Prepronociceptin | PNOC | high |
| 1911.54 | 1910 | 0.28(-0.13-1.65) | 1.13(0.40-2.13) | 0.02039 | Immunoglobulin heavy joining 1 | IGHJ1 | low |
| 2022.01 | 2021 | 1.18(0.15-2.17) | 0.56(-0.26-0.93) | 0.006902 | Complement C3 | C3 | high |
| 2341.91 | 2343 | 0.57(0.01-1.81) | 0.25(-0.11-0.46) | 0.020633 | Prosalusin | TOR2A | high |
| 2549.39 | 2550 | 0.75(0.12-1.91) | 0.11(-0.32-0.56) | 0.000308 | Chromogranin-A | CHGA | high |
| 2628.18 | 2630 | 0.56(0.05-1.59) | 0.27(-0.04-0.55) | 0.026034 | Integral membrane protein 2B | ITM2B | high |
| 2662.54 | 2661 | 0.64(0.02-1.61) | 0.27(-0.04-0.71) | 0.04659 | Humanin-like 8 | MTRNR2L8 | high |
| 2737.31 | 2737 | 1.15(0.24-2.66) | 4.33(1.89-10.35) | 1.13E-06 | Humanin-like 11 | MTRNR2L11 | low |
| 2878.04 | 2876 | 0.60(0.07-1.36) | 0.19(-0.10-0.66) | 0.020151 | Presenilins-associated rhomboid-like protein, mitochondrial | PARL | high |
| 3055.01 | 3054 | 0.35(-0.04-1.08) | 0.10(-0.32-0.47) | 0.018763 | B melanoma antigen 1 or B melanoma antigen 5 | BAGE or BAGE5 | high |
| 3079.82 | 3079 | 0.41(-0.07-1.13) | -0.06(-0.23-0.21) | 0.001839 | Neuropeptide B | NPB | high |
| 3297.52 | 3299 | 0.77(0.20-1.66) | 1.43(0.65-4.87) | 0.005263 | Glucagon | GCG | low |
| 3822.20 | 3820 | 0.54(0.08-1.32) | 0.09(-0.18-0.52) | 0.002301 | Putative gastric cancer-related gene 224 protein | GCRG224 | high |
| 3988.30 | 3985 | 0.56(0.14-1.20) | 0.25(0.04-0.44) | 0.028195 | Proenkephalin-B | PDYN | high |
| 4017.60 | 4021 | 0.59(0.13-1.51) | 0.20(0.00-0.47) | 0.002951 | Vasopressin-neurophysin 2-copeptin | AVP | high |
| 4038.06 | 4035 | 0.74(0.16-1.42) | 0.36(0.01-0.87) | 0.034852 | Coatomer subunit alpha | COPA | high |
| 4054.55 | 4055 | 0.87(0.24-1.30) | 0.19(-0.12-0.65) | 0.000447 | Putative DNA-binding protein inhibitor ID-2B | ID2B | high |
| 4184.52 | 4183 | 0.74(0.20-1.41) | 0.21(-0.16-0.49) | 0.000359 | Pancreatic prohormone | PPY | high |
| 4262.02 | 4260 | 0.98(0.25-2.40) | 0.46(0.27-1.01) | 0.018101 | Parathyroid hormone-related protein | PTHLH | high |
| 4278.20 | 4276 | 0.84(0.27-1.71) | 0.30(-0.01-0.86) | 0.00109 | Defensin-5 | DEFA5 | high |
| 4367.30 | 4371 | 0.62(0.08-1.18) | 0.33(0.03-0.57) | 0.033719 | Salivary acidic proline-rich phosphoprotein 1/2 | PRH1 or PRH2 | high |
| 4426.51 | 4424 | 0.58(0.16-1.19) | 0.26(0.04-0.35) | 0.005336 | Cocaine- and amphetamine-regulated transcript protein | CARTPT | high |
| 4756.84 | 4759 | 0.71(0.26-1.72) | 0.45(0.20-0.74) | 0.028515 | Corticoliberin | CRH | high |
| 5553.90 | 5552 | 0.26(-0.02-0.57) | -0.02(-0.11-0.18) | 0.001705 | Protransforming growth factor alpha | TGFA | high |
| 5591.43 | 5590 | 0.28(0.07-0.55) | 0.19(-0.04-0.26) | 0.00672 | Basic salivary proline-rich protein 1 or Basic salivary proline-rich protein 2 | PRB1 or PRB2 | high |
| 5876.83 | 5874 | 0.38(0.15-0.80) | 0.11(-0.04-0.30) | 0.000371 | 3-hydroxy-3-methylglutaryl-CoA lyase, cytoplasmic or Putative uncharacterized protein C20orf187 | HMGCLL1 or C20orf187 | high |
| 6043.83 | 6042 | 0.44(0.14-0.81) | 1.81(1.10-3.22) | 4.92E-09 | Metallothionein-2 | MT2A | low |
| 6199.08 | 6202 | 0.50(0.24-1.01) | 0.90(0.50-1.54) | 0.021882 | Keratin-associated protein 20-1 | KRTAP20-1 | low |
| 6359.66 | 6362 | 0.29(0.10-0.63) | 0.14(0.05-0.28) | 0.019678 | Cytochrome c oxidase subunit 7B, mitochondrial | COX7B | high |
| 6782.83 | 6784 | 0.24(0.04-0.52) | 0.14(0.01-0.20) | 0.044665 | Cytochrome c oxidase copper chaperone | COX17 | high |
| 6944.25 | 6950 | 0.22(0.06-0.55) | 0.01(-0.08-0.18) | 0.000341 | Hypoxia-inducible lipid droplet-associated protein | HILPDA | high |
| 7204.07 | 7201 | 0.28(0.10-0.48) | 0.09(-0.08-0.25) | 0.001142 | Serine protease inhibitor Kazal-type 7 | SPINK7 | high |
| 7796.58 | 7798 | 0.26(0.09-0.48) | 0.35(0.24-0.56) | 0.039714 | C-C motif chemokine 3-like 1 | CCL3L1 | low |
| 7834.01 | 7841 | 0.40(0.15-0.74) | 1.44(1.13-1.71) | 1.50E-10 | 40S ribosomal protein S28 | RPS28 | low |
| 8323.92 | 8316 | 0.26(0.06-0.49) | 0.08(-0.06-0.21) | 0.001604 | C-X-C motif chemokine 6 | CXCL6 | high |
| 8374.59 | 8374 | 0.25(0.09-0.48) | 0.08(-0.07-0.20) | 0.000756 | Ankyrin-1 | ANK1 | high |
| 8504.70 | 8510 | 0.17(0.05-0.33) | 0.08(-0.01-0.13) | 0.003712 | Putative uncharacterized protein encoded by LINC00313 | LINC00313 | high |
| 8654.96 | 8654 | 0.33(0.13-0.58) | 0.49(0.31-0.67) | 0.026942 | Ly6/PLAUR domain-containing protein 2 | LYPD2 | low |
| 8802.89 | 8800 | 0.17(0.03-0.35) | 0.45(0.19-0.61) | 0.000264 | C-C motif chemokine 19 | CCL19 | low |
| 8867.85 | 8865 | 0.27(0.11-0.55) | 2.11(1.20-3.37) | 6.99E-13 | Platelet basic protein | PPBP | low |
| 9105.42 | 9106 | 0.16(0.03-0.35) | 0.44(0.24-0.68) | 0.00015 | Platelet basic protein or Keratin-associated protein 19-4 | PPBP or KRTAP19-4 | low |
| 9493.79 | 9496 | 0.21(0.12-0.41) | 1.18(0.88-1.76) | 8.86E-12 | C-C motif chemokine 25 or Cytochrome c oxidase subunit 6A2, mitochondrial | CCL25 or COX6A2 | low |
| 9536.92 | 9539 | 0.24(0.10-0.54) | 4.91(2.92-6.65) | 1.75E-13 | High mobility group nucleosome-binding domain-containing protein 4 | HMGN4 | low |
| 9635.81 | 9638 | 0.25(0.10-0.49) | 1.53(1.12-2.49) | 1.45E-11 | COMM domain-containing protein 6 | COMMD6 | low |
| 9771.01 | 9775 | 0.27(0.13-0.57) | 1.03(0.85-1.53) | 9.67E-07 | Cytochrome b-c1 complex subunit 8 | UQCRQ | low |
| 9832.62 | 9828 | 0.20(0.10-0.47) | 0.67(0.51-0.99) | 1.53E-05 | Caspase-9 | CASP9 | low |
| 10782.9 | 10785 | 0.17(0.07-0.39) | 0.07(0.01-0.19) | 0.006033 | Immunoglobulin heavy variable 4-31 or T cell receptor delta variable 2 | IGHV4-31 or TRDV2 | high |
| 10853.9 | 10853 | 0.18(0.04-0.36) | 0.06(0.01-0.11) | 0.000664 | Immunoglobulin heavy variable 3-7 or Uncharacterized protein EXOC3-AS1 | IGHV3-7 or EXOC3-AS1 | high |
| 10968.2 | 10968 | 0.17(0.04-0.42) | 0.06(0.03-0.12) | 0.002665 | Prostate collagen triple helix protein | PCOTH | high |
| 11660.4 | 11662 | 0.62(0.21-1.50) | 0.23(0.13-0.31) | 3.18E-05 | Protein S100-A14 | S100A14 | high |
| 11742.4 | 11741 | 0.71(0.31-2.03) | 0.12(0.08-0.23) | 3.56E-09 | Protein S100-A11 | S100A11 | high |
| 11904.6 | 11906 | 0.40(0.21-1.01) | 0.13(0.03-0.19) | 5.99E-07 | Natriuretic peptides B | NPPB | high |
| 13297.8 | 13299 | 0.12(0.03-0.22) | 0.05(0.01-0.08) | 0.003127 | Zinc-activated ligand-gated ion channel or Uncharacterized protein encoded by LINC01561 or 5'(3')-deoxyribonucleotidase, cytosolic type | ZACN or LINC01561 or NT5C | high |
| 13361.8 | 13361 | 0.11(0.02-0.23) | 0.06(0.01-0.09) | 0.006033 | Putative uncharacterized protein encoded by LINC00518 | LINC00518 | high |
| 13586.4 | 13584 | 0.18(0.06-0.37) | 0.07(0.04-0.09) | 0.000225 | Teratocarcinoma-derived growth factor 1 | TDGF1 | high |
| 13892.0 | 13891 | 0.11(0.04-0.22) | 0.04(0.03-0.09) | 0.004845 | Pleckstrin homology-like domain family A member 3 | PHLDA3 | high |
| 14362.9 | 14363 | 0.10(0.03-0.21) | 0.05(0.02-0.09) | 0.005191 | 2-iminobutanoate/2-iminopropanoate deaminase or V-type proton ATPase subunit G 3 or BRCA1-associated RING domain protein 1 | RIDA or ATP6V1G3 or BARD1 | high |
| 15483.2 | 15483 | 0.14(0.06-0.21) | 0.07(0.03-0.11) | 0.02458 | Uncharacterized protein C1orf53 | C1orf53 | high |
| 15695.9 | 15696 | 0.08(0.04-0.17) | 0.04(0.00-0.10) | 0.018988 | 15-hydroxyprostaglandin dehydrogenase [NAD(+)] | HPGD | high |
| 15909.1 | 15909 | 0.11(0.03-0.20) | 0.03(0.02-0.06) | 0.00051 | Membrane-spanning 4-domains subfamily A member 6E | MS4A6E | high |
